# Distinct Roles for Dectin-1 and TLR4 in the Pathogenesis of *Aspergillus fumigatus* Keratitis

**DOI:** 10.1371/journal.ppat.1000976

**Published:** 2010-07-01

**Authors:** Sixto M. Leal, Susan Cowden, Yen-Cheng Hsia, Mahmoud A. Ghannoum, Michelle Momany, Eric Pearlman

**Affiliations:** 1 Department of Ophthalmology and Visual Sciences, Case Western Reserve University, Cleveland, Ohio, United States of America; 2 Department of Pathology, Case Western Reserve University, Cleveland, Ohio, United States of America; 3 Department of Plant Biology, University of Georgia, Athens, Georgia, United States of America; 4 Center for Medical Mycology, Case Western Reserve University, Cleveland, Ohio, United States of America; UMass Medical Center, United States of America

## Abstract

*Aspergillus* species are a major worldwide cause of corneal ulcers, resulting in visual impairment and blindness in immunocompetent individuals. To enhance our understanding of the pathogenesis of *Aspergillus* keratitis, we developed a murine model in which red fluorescent protein (RFP)-expressing *A. fumigatus* (Af293.1RFP) conidia are injected into the corneal stroma, and disease progression and fungal survival are tracked over time. Using Mafia mice in which *c-fms* expressing macrophages and dendritic cells can be induced to undergo apoptosis, we demonstrated that the presence of resident corneal macrophages is essential for production of IL-1β and CXCL1/KC, and for recruitment of neutrophils and mononuclear cells into the corneal stroma. We found that β-glucan was highly expressed on germinating conidia and hyphae in the cornea stroma, and that both Dectin-1 and phospho-Syk were up-regulated in infected corneas. Additionally, we show that infected Dectin-1^−/−^ corneas have impaired IL-1β and CXCL1/KC production, resulting in diminished cellular infiltration and fungal clearance compared with control mice, especially during infection with clinical isolates expressing high β-glucan. In contrast to Dectin 1^−/−^ mice, cellular infiltration into infected TLR2^−/−^, TLR4^−/−^, and MD-2^−/−^ mice corneas was unimpaired, indicating no role for these receptors in cell recruitment; however, fungal killing was significantly reduced in TLR4^−/−^ mice, but not TLR2^−/−^ or MD-2^−/−^ mice. We also found that TRIF^−/−^ and TIRAP^−/−^ mice exhibited no fungal-killing defects, but that MyD88^−/−^ and IL-1R1^−/−^ mice were unable to regulate fungal growth. In conclusion, these data are consistent with a model in which β-glucan on *A.fumigatus* germinating conidia activates Dectin-1 on corneal macrophages to produce IL-1β, and CXCL1, which together with IL-1R1/MyD88-dependent activation, results in recruitment of neutrophils to the corneal stroma and TLR4-dependent fungal killing.

## Author Summary

Corneal infection with filamentous fungi, including *Aspergillus* species, is a common cause of visual impairment and blindness in the southern USA and worldwide. The incidence in India and China greatly increases during harvest season when infection occurs after traumatic injury with fungal spores (conidia). In contrast to pulmonary aspergillosis, keratitis occurs in immunocompetent individuals. To characterize the host response, we injected *Aspergillus fumigatus* conidia expressing red fluorescence into the transparent mouse cornea, and showed that cytokine production, neutrophil and monocyte recruitment to the corneal stroma and fungal killing is dependent on the presence of macrophages and dendritic cells, and on expression of the β-glucan receptor Dectin-1. We also found that fungal killing, but not cellular infiltration, is dependent on expression of the LPS receptor TLR4. In addition, we demonstrate that IL-1R1 and MyD88 regulate neutrophil recruitment and fungal killing in *Aspergillus* keratitis, whereas TLR4 associated adaptor molecules TRIF and TIRAP have no role. Together, these findings identify specific mediators of the innate immune response to these organisms that regulate disease severity and survival of *Aspergillus* that may have potential application as targets for therapeutic intervention.

## Introduction

Fungal infections of the cornea (i.e. fungal keratitis) account for approximately 1% of corneal ulcers in temperate regions of industrialized nations [1]. However, in tropical regions of developed countries, such as in the southeastern United States, fungal infections of the cornea account for up to 35% of all corneal ulcers resulting in severe visual impairment and blindness [1]–[3]. Globally, the impact of fungal keratitis on visual health is even greater, with reports of up to 60% of corneal ulcers attributable to fungal infection in developing nations including China, Nepal, India, Bangladesh, Ghana and Mexico [4]–[11]. The etiological agents of these corneal infections are most commonly filamentous *Fusarium* (*F.solani*, *F.oxysporum*) [1], [12], and *Aspergillus* species (*A.flavus*, *A. fumigatus*, *nomius* [13], *A.tamarii* [14], *A. terreus*, and *A.tubingensis* [15], [16]), which are prevalent in hot, humid climates, and where the predominant risk factor is traumatic injury associated with agricultural work.

As *Aspergillus* is ubiquitous in the environment, the population at large is constantly exposed to this opportunistic pathogen [17]–[19], and conidia are inhaled at an estimated 200 conidia per day [19]–[21]. In areas of high endemicity, *A. fumigatus* can also be isolated from the conjunctival sac of healthy individuals [22]. Thus, in the setting of a disrupted corneal epithelium, there is broad opportunity for inoculation of *A. fumigatus* conidia (2–3 µm diameter) or conidiophores (>100 individual conidia per hyphal stalk) from airborne vegetative matter or the conjunctiva into the corneal stroma in association with traumatic injury [8], [23]. Subsequently, fungal virulence factors that facilitate hyphal invasion of the tissue (e.g. toxin and protease secretion) [24]–[29], together with low efficacy of anti-mycotic therapy [30]–[32], and the resulting inflammatory response all converge to induce destruction of corneal tissue. Treatment failure also occurs in up to 60% of patients, who may require at least one and sometimes repeated corneal transplantation, and in severe cases results in enucleation of the infected eye [23], [31]. In contrast to pulmonary aspergillosis, which is associated with immune suppression, fungal keratitis occurs in otherwise healthy, immunocompetent individuals.

Despite the impact of filamentous fungal infections on global blindness, and a recent outbreak of contact lens-associated *Fusarium* keratitis in the USA [12], the pathogenesis of this disease is not well understood. We therefore developed a murine model of corneal infection using *Aspergillus* that constitutively express dsRed fluorescent protein at each stage of development, and are therefore readily detected in the transparent cornea. Our findings demonstrate that Dectin-1 mediates the initial wave of pro-inflammatory and chemotactic cytokine production and cellular infiltration into the cornea. We also show that the IL-1R1/MyD88 pathway further propagates cellular recruitment into the cornea during *Aspergillus* infection, and that in contrast to Dectin 1, TLR4 does not regulate cellular infiltration, but is essential for anti-fungal activity.

## Results

### Development of a murine model of *Aspergillus* keratitis

**Figure 1:**
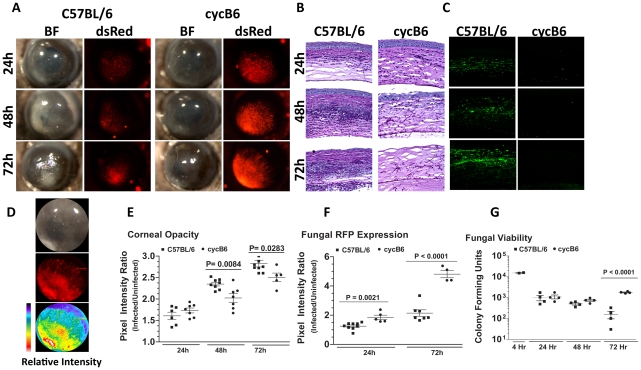
*Aspergillus fumigatus* keratitis in cyclophosphamide treated mice. A mouse model of *Aspergillus* keratitis was developed by injecting 1×10^5^ Af293.1RFP conidia into the corneal stroma, and tracking disease progression over time. **A.** Slit-lamp microscopy of immunocompetent and cyclophosphamide-immunosuppressed (Cyc) C57BL/6J corneas infected for 24, 48, and 72h. Fluorescence microscopy of emitted 580nm light reveals fungal growth in the corresponding cornea. **B.** 5µm paraffin section of the central cornea were stained with Periodic Acid Schiff and Hematoxylin (PASH) to visualize cellular infiltration, fungal growth, and corneal pathology at 24, 48, and 72 h post-infection. **C.** 5µm paraffin sections were immunostained with anti-neutrophil NIMPR-14 to visualize neutrophil infiltration at 24, 48, and 72 h post-infection. **D.** Bright-light reflected from the cornea, and 580nm light emitted from the cornea were independently captured as TIFF images and the pixel intensity of each image was analyzed within a constant defined region encompassing the cornea to quantify corneal opacity or fungal RFP expression (corresponding to fungal biomass), respectively. **E.** Corneal opacity in immunocompetent and cyc-immunosuppressed C57BL/6 mice at 24–72h post-infection **F.** Fungal RFP expression in immunocompetent and cyc-immunosuppressed C57BL/6 mice at 24 and 72h post-infection. **G.** At each time point, eyes were enucleated, homogenized, and fungal viability assessed via serial dilutions on Sabouraud Dextrose agar. Data are representative of two independent experiments, with five mice per time point.

To visualize *A.fumigatus* during infection of the transparent mammalian cornea, we generated a monomeric dsRED RFP-expressing *A. fumigatus* strain. **Figure S1** illustrates the structure of the plasmid, and shows expression of RFP at each lifecycle stage of *Aspergillus*. To determine the role of the host response to these organisms in the cornea, C57BL/6 mice were either treated systemically with cyclophosphamide (cyc), or were left untreated prior to injecting 1×10^5^ Af293.1RFP conidia into the corneal stroma (Preliminary studies revealed that 1×10^5^ conidia was the smallest inoculum in which organisms could be recovered from the cornea after 24h). Corneal opacification, fungal growth, survival, and cellular infiltration were assessed at each time point. **Figure 1A** shows that at 24h post-infection, immunocompetent C57BL/6 mice developed significant corneal opacity, which peaked at 48h, and persisted up to 72h post-infection. However, cyclophosphamide-treated mice had significantly lower corneal opacification scores than immunocompetent mice at 48 and 72h post-infection.

To characterize the host response in *Aspergillus* keratitis, 5µm corneal sections were stained with PASH and examined by brightfield microscopy. As shown in **Figure 1B**, there was a pronounced cellular infiltration in the corneas of immunocompetent mice between 24 and 72h, with neutrophils also present in the anterior chamber (for comparison, a PASH stained section of a normal mouse cornea is shown in **Supplementary Figure S2A**). In contrast, there was less cellular infiltration in corneas of cyclophosphamide-treated mice at each time point. There was also enhanced fungal penetration though Descemet's membrane and into the anterior chamber at 48 and 72h post-infection, and increased epithelial debridement in cyclophosphamide treated mice compared with immunocompetent mice. **Figure 1C** shows that cells in the C57BL/6 cornea are NIMP-R14^+^ neutrophils, and that the numbers increased over time. Neutrophils were not detected in corneas of cyclophosphamide-treated mice at any time post-infection, and in contrast to immunocompetent mice, longer-term infection of treated mice results in corneal perforation (data not shown).

To quantify corneal opacification and hypertrophic and filamentous fungal growth during corneal infection, we used image analysis software that provides numerical output for corneal opacification and for fungal dsRed expression for all animals in the experiment rather than representative mice. **Figure 1D** shows a representative image analysis derivation, which shows color (pixel) intensity. Using this method, we found that opacification was significantly lower in cyclophosphamide treated animals (**Figure 1E**). Conversely, fungal dsRed expression was significantly elevated in corneas of cyclophosphamide-treated mice compared with immunocompetent mice at each time point (**Figure 1F**). There was no detectable red fluorescence in uninfected mice (**Figure S2**). To examine fungal viability, eyes were homogenized, and the number of colony forming units (CFU) was determined by standard methods. **Figure 1G** depicts a 1 log-fold reduction in fungal CFU in immunocompetent mice between 48h and 72h post-infection, whereas CFU were not reduced in cyclophosphamide-treated C57BL/6 corneas over this time period.

Together, these findings identify a resistant phenotype in immunocompetent animals, where there is a pronounced neutrophil infiltration to the corneal stroma, resulting in controlled fungal growth. These findings also show a susceptible phenotype illustrated by cyclophosphamide treated mice, where neutrophil recruitment to the corneal stroma is impaired, fungal growth continues unabated, and the cornea eventually perforates.

### Resident corneal c-fms^+^ macrophages and dendritic cells mediate cellular recruitment during *Aspergillus* keratitis

The predominant cells in the corneal stroma are keratocytes, which produce collagen and proteoglycans that comprise the extracellular matrix; however there is also a population of resident macrophages and dendritic cells at this site [33], [34]. To determine if resident macrophages and dendritic cells mediate the initial recognition of *A. fumigatus* in the cornea, we utilized Macrophage Fas Induced Apoptosis (Mafia) mice, which express eGFP and a membrane bound suicide protein under control of the myeloid-lineage specific *c-fms* promoter [35], [36]. In these mice, all macrophages and dendritic cells express eGFP constitutively, and undergo cell-lineage specific apoptosis after cross-linking the FK506 binding domain of the membrane-bound suicide protein using the FK506 dimerizer AP20187 [36].

**Figure 2:**
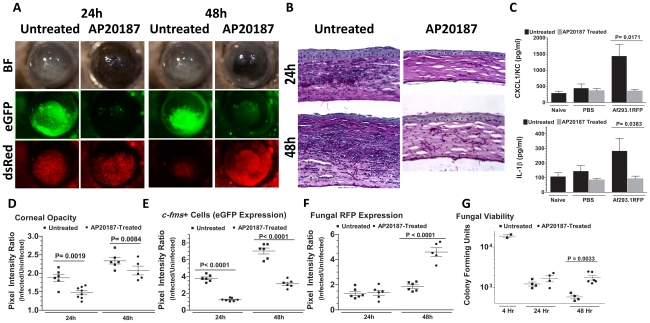
The role of macrophages in *A. fumigatus* keratitis. Mafia mice were used to identify the resident corneal cell-types responsible for recognizing *A. fumigatus* and initiating cellular recruitment into the cornea **A.** Brightfield microscopy of Mafia mice +/− 5 day AP20187- dimerizer treatment at 24 and 48h post-infection. 488 nm fluorescence images indicate monocytic recruitment into the cornea and 580nm fluorescence indicates fungal RFP expression. **B.** 5µm sections of the central cornea at 24 and 48h post-infection were PASH stained and used to visualize cellular infiltration, fungal growth, and corneal pathology in treated vs. untreated Mafia mice. **C.** ELISA analysis at 10h post-infection in treated vs. untreated Mafia mice for CXCL1/KC and IL-1β. **D.** Corneal opacity in treated vs. untreated Mafia mice was objectively quantified using Metamorph software. **E.** *c-fms* ^+^ cell eGFP expression and **F.** Fungal RFP expression were quantified at 24 and 48h post-infection. **G.** Eyes were enucleated, homogenized and subjected to serial plating and CFU quantification. Data are representative of two independent experiments, with five mice per time point.

We showed previously that eGFP^+^ macrophages/dendritic cells are readily identified in the corneas of naïve untreated Mafia mice, and that eGFP expressing cells are depleted in AP20187-treated Mafia mice [35]. To ascertain the role of macrophages and dendritic cells in *Aspergillus* keratitis, Mafia mice were treated with AP20187, and infected with 1×10^5^ Af293.1RFP conidia. Corneas of untreated Mafia mice developed opacification at 24 and 48h after infection, consistent with increased eGFP^+^ cellular infiltration to the corneal stroma and dsRed expressing *Aspergillus* hyphae (**Figure 2A**). In contrast, corneas of AP20187-treated Mafia mice had decreased opacification, and significantly increased dsRed expressing *Aspergillus* hyphae (**Figures 2A**). Cellular infiltration to the corneal stroma was absent in infected AP20187-treated Mafia mice as shown by the absence of eGFP^+^ cells (**Figure 2A**) and in histological sections (**Figure 2B**).

To determine if the impaired cellular infiltration is related to pro-inflammatory and chemotactic cytokine production, corneas were dissected from untreated and AP20187-treated Mafia mice at 10h after intrastromal injection of Af293.1RFP conidia or PBS (trauma control), and prior to detectable cellular infiltration. Corneas were homogenized, and CXCL1/KC and IL-1β production were examined by ELISA. **Figure 2C** shows that both CXCL1/KC and IL-1β were elevated in untreated, but not AP20187-treated corneas from Mafia mice.

**Figures 2D–F** show image analysis based quantification of corneal opacification, eGFP+ cell infiltration, and RFP expression for individual corneas in the experiment and reveal significantly lower corneal opacification (**Figure 2D**) and eGFP^+^ cell infiltration (**Figure 2E**) in AP20187-treated Mafia mice. Conversely, dsRed hyphae (**Figure 2E**) and CFU (**Figure 2F**) were significantly higher in corneas of AP20187-treated Mafia mice compared with untreated Mafia mice, indicating that *cfms^+^* cells regulate fungal growth and survival (**Figure 2G**).

Taken together, these data show that resident *c-fms* ^+^ macrophages and dendritic cells in the naïve cornea produce IL-1β and CXCL1/KC, which likely mediate neutrophil recruitment into the cornea, and subsequently limit *Aspergillus* growth and survival.

### Spleen tyrosine kinase (Syk) is phosphorylated during *Aspergillus* keratitis

**Figure 3:**
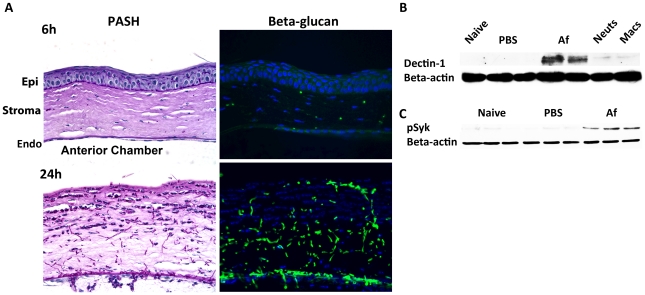
β-glucan expression and Dectin-1 signaling in *A. fumigatus* keratitis. Fungal β-glucan expression and Dectin-1 protein activation in the cornea during *Aspergillus* infection were assessed by IHC and western blot analysis, respectively. **A.** 5µm central cornea sections at 6 and 24h post-infection were PASH stained to visualize swollen conidia (6h) and hyphae (24h), while β-glucan expression was detected using a mouse anti-fungal β-glucan antibody. B. Western blot analysis using anti-Dectin-1 antibody or **C.** anti-pSyk antibody was performed on cornea protein lysates from naïve, 10h PBS injected (trauma control), and 10hr *A. fumigatus* infected corneas. Data are representative of two independent experiments, with three corneas per time point. [Endo- endothelium Epi- epithelium, Neuts- casein-elicited peritoneal neutrophils. MACs- thioglycolate-elicited peritoneal, macrophages.]

As resident *cfms* ^+^ macrophages and dendritic cells are essential for early cytokine production in the cornea after *Aspergillus* infection, they are likely the first cells to respond to germinating conidia. We therefore examined the pathogen recognition molecules initiating this response, including Dectin-1, TLR2 and TLR4. Dectin-1 is a C-type lectin expressed by myeloid-derived cells that recognizes β-glucan when it is exposed on the cell wall, and β-glucan expression in *Aspergillus* occurs in germinating, but not dormant conidia, and in hyphal stages [37]–[39]. We examined β-glucan and Dectin-1 expression and activation in the cornea after *Aspergillus* infection. As shown in **Figure 3A**, PASH stained corneas revealed swollen conidia (6h) and hyphae (24h); further, β-glucan expression was apparent in both forms, especially at 24h when hyphal forms predominated, indicating that *Aspergillus* expresses the ligand for Dectin-1 during corneal infection.

To determine the effect of *Aspergillus* on Dectin-1 protein levels in the cornea, we dissected corneas 10h after infection or after injection with PBS (trauma controls), and processed the corneas for western blot analysis. Dectin-1 was not detected in naïve and PBS-injected corneas; however, as early as 10h after *Aspergillus* infection, Dectin-1 was clearly expressed in the cornea (**Figure 3B**) Further, phosphorylated Spleen Tyrosine Kinase (Syk) was elevated in infected corneas, compared with naïve and trauma controls (**Figure 3C**). Given that Syk phosphorylation is indicative of Dectin-1 activation [40], these findings indicate that not only is Dectin-1 expressed in the cornea, but that it is also activated during *Aspergillus* infection.

### Dectin-1 mediates cytokine production and cellular infiltration after infection with Af293.1RFP

**Figure 4:**
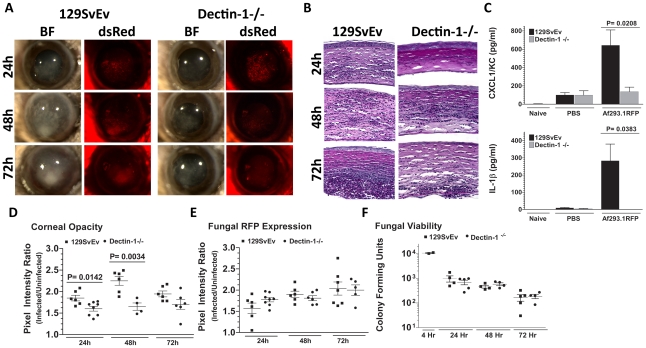
Role of Dectin-1 in keratitis caused by *A. fumigatus* Af293.1RFP. To determine the role of Dectin-1 in recognizing *A. fumigatus* and initiating cellular recruitment into the cornea, we injected 1×10^5^ Af293.1RFP conidia into the stroma of 129SvEv and Dectin-1^−/−^ mice. **A.** Brightfield and fluorescence microscopy of corneas from 129SvEv and Dectin-1^−/−^ mice at 24, 48, and 72h post-infection. **B.** 5µm PASH stained central cornea sections of infected 129SvEv and Dectin-1^−/−^ mice at 24, 48, 72h reveal the extent of cellular infiltration, fungal growth, and corneal pathology in the absence of Dectin-1. **C.** ELISA analysis at 10h post-infection in 129SvEv vs. Dectin-1^−/−^ mice for CXCL1/KC and IL-1β. **D.** Corneal opacity, **E.** Fungal RFP expression, and **F.** Fungal viability at set time-points post-infection. Data is representative of 5 independent experiments, 5 mice per time point.

As we found β-glucan expression *in vivo*, and increased Dectin-1/pSyk activation in infected corneas, we next examined the role of Dectin-1 during *Aspergillus* keratitis. Dectin-1^−/−^ and control 129SvEv mice were injected intrastromally with Af293.1RFP conidia, and corneal opacification, cellular infiltration, and fungal survival were measured as before. As shown in **Figure 4A**, corneal opacification was evident in 129SvEv corneas by 24h post-infection, increased at 48h and decreased at 72h, whereas corneal opacification was significantly lower in Dectin-1^−/−^ mice at 24h and 48h. However, there were no significant differences between Dectin-1^−/−^ and control, 129SvEv corneas in fungal dsRed expression (**Figure 4A**). To determine the role of Dectin-1 in cytokine production and cellular infiltration to the cornea, Dectin-1^−/−^ and 129SvEv mice were infected as before, eyes were processed for histology, and 5µm sections were stained with PASH. As shown in **Figure 4B**, cellular infiltration in 129SvEv mice was similar to infected C57BL/6 mice, with pronounced cellular infiltration at each time point. In contrast, Dectin-1^−/−^ corneas had impaired cellular infiltration at each time point. To examine if the decreased cellular infiltration in Dectin-1^−/−^ mice was associated with pro-inflammatory and chemotactic cytokine production, corneas were dissected and homogenized at 10h post-infection (prior to detectable cellular infiltration), and CXCL1/KC and IL-1β were measured by ELISA. Consistent with impaired cellular infiltration, infected Dectin-1^−/−^ corneas had significantly less CXCL1/KC and IL-1β compared with 129SvEv mice (**Figure 4C**).

Quantification by image analysis shows elevated corneal opacification in 129SvEv compared with Dectin-1^−/−^ mice 24h and 48h after infection (**Figures 4D**), but no difference in fungal RFP expression (**Figures 4E**). Consistent with the latter observation, there were no differences in fungal CFUs between Dectin-1^−/−^ and 129SvEv mice at any time point examined (**Figure 4F**).

These findings demonstrate that Dectin-1 regulates cytokine production in the cornea, cellular infiltration to the corneal stroma and development of corneal opacification; however, Dectin-1 expression was not required to regulate growth and survival of strain Af293.1RFP.

### Dectin-1 regulates growth and survival of high β-glucan expressing *A.fumigatus* clinical isolates

**Figure 5:**
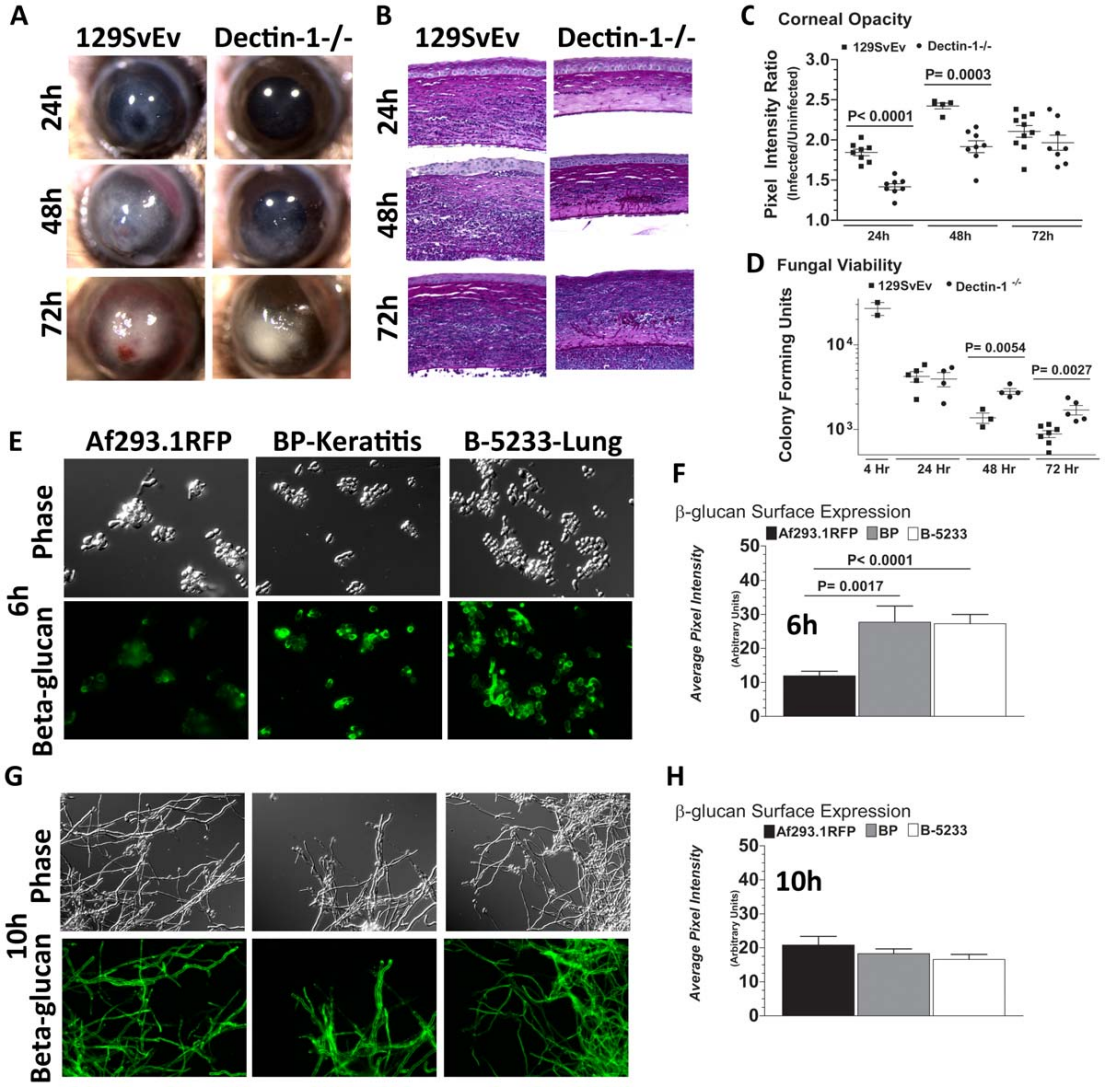
Role of Dectin-1 in keratitis caused by a clinical isolate of *A. fumigatus*. To determine the role of Dectin-1 in *Aspergillus* keratitis during infection with a clinical isolate we injected 1×10^5^ conidia from Strain Af-BP into the corneal stroma and tracked disease progression over time. **A.** Brightfield microscopy of corneas from 129SvEv and Dectin-1^−/−^ mice at 24, 48, and 72h post-infection **B.** 5µm PASH stained central cornea sections of infected 129SvEv and Dectin-1^−/−^ mice **C.** Corneal opacity, and **D.** Fungal viability at set time-points post-infection. Data is representative of 2 independent experiments, 10 mice per time point. **E,G.** Mouse anti-fungal β-glucan IgM was used to detect β-glucan surface expression in *A. fumigatus* swollen conidia/germlings (Strains Af293.1RFP, Af-BP-KO, B-5233) grown for 6h (E), or 10h (G) in SDA media. **F,H.** Quantification of β-glucan surface expression in Strains Af293.1RFP, Af-BP-KO, and B-5233 grown for 6h (F) and 10h (H) in SDA media. β-glucan expression was determined using Metamorph software. Data are representative of two independent experiments.

Failure to detect increased survival of Af293.1RFP in Dectin-1^−/−^ mice, despite decreased cellular infiltration at all stages post-infection, led us to hypothesize that fungal survival in Dectin-1^−/−^ mice is dependent on the virulence of the infecting *A.fumigatus* strain. We therefore infected Dectin-1^−/−^ and 129SvEv mice with a clinical isolate from a patient with fungal keratitis. Injection of Strain Af-BP into the 129SvEv cornea induced corneal opacification at 24h post-infection, which increased after 48 and 72h (**Figure 5A**). In contrast to strain Af293.1RFP, the Af-BP isolate also caused engorgement of limbal blood vessels after 48h, and hemorrhage after 72h post-infection (**Figure 5A**), and may be reflective of the characteristic angio-invasiveness of clinical *A.fumigatus* isolates [41]. Dectin-1^−/−^ mice exhibited impaired cellular infiltration (**Figure 5B**), and had significantly lower corneal opacification at 24 and 48h post-infection with Strain Af-BP (**Figure 5C**), which was similar to infection with strain Af293.1RFP. However, in contrast to infection with Af293.1RFP, fungal CFUs recovered from infected corneas were significantly elevated in Dectin-1^−/−^ compared with 129SvEv mice at 48 and 72h post-infection (**Figure 5D**). There were no apparent structural differences between naïve Dectin-1^−/−^ and wild type corneas (data not shown).

To determine if Dectin-1 regulation of Af-BP but not Af293.1RFP survival is related to β-glucan surface expression, we cultured strains Af293.1RFP, Af-BP, and the lung isolate B-5233 in SDA media for 6h or 10h, and examined β-glucan expression as described above. β-glucan was not detected on resting conidia of Af293.1RFP or the clinical isolates (data not shown). However, at 6 h (swollen and germinating conidia), there was significantly higher surface β-glucan expression in the clinical isolates Af-BP and B-5233 compared with laboratory strain Af293.1RFP (**Figure 5E, F**). β-glucan surface expression on hyphae after 10h growth was also quantified; as shown in **Figure 5G,H**, there were no significant differences among these strains at this time point. These findings indicate that strains with high surface β-glucan expression on germinating conidia are more likely to be detected by Dectin-1 and to induce a more pronounced cellular infiltrate, resulting in increased fungal killing.

### Dectin-1 regulates *A.fumigatus* activation of bone marrow macrophages

Since macrophage depleted Mafia mice and Dectin-1^−/−^ mice exhibited impaired cellular recruitment to the cornea during *Aspergillus* infection, we next examined the role of Dectin-1 in activation of bone marrow derived macrophages (BMMs) by *A.fumigatus*. Af-BP swollen conidia were isolated after 6h incubation with Sabouraud dextrose media, fixed, and incubated with 129SvEv or Dectin-1^−/−^ BMMs at an MOI = 100. After 15, 30, and 60 min, cells were lysed, and Syk and Iκb phosphorylation were detected by western blot analysis, and NFκB nuclear translocation was examined after incubation with anti-p65 antibody.

**Figure 6:**
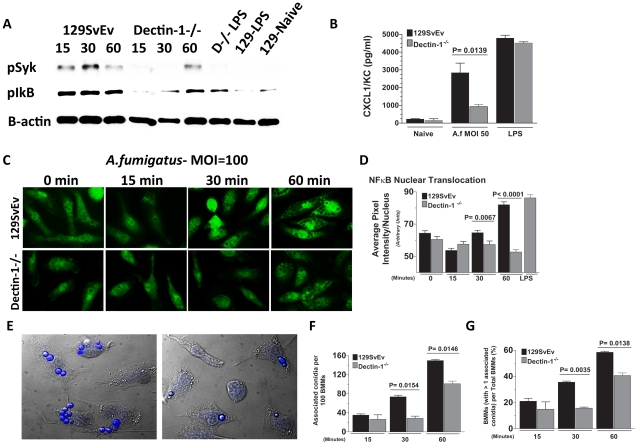
Role of Dectin-1 in activation of bone marrow-derived macrophages. Bone marrow-derived macrophages (BMM) from 129SvEv and Dectin-1^−/−^ mice were incubated with *A. fumigatus* swollen conidia Strain Af-BP (MOI = 100). **A.** Protein lysates from 0, 15, 30, and 60 min stimulated 129SvEv and Dectin-1^−/−^ BMMs were examined via western blot analysis, using anti-pSyk and anti-pIκB antibodies. **B.** 129SvEv and Dectin-1^−/−^ BMM culture supernatants were harvested at 60 min post conidia exposure and CXCL1/KC levels assessed via ELISA. **C.** 129SvEv and Dectin-1^−/−^ BMMs were seeded onto coverslips, and exposed to conidia for 0, 15, 30, and 60 min. Subsequently, BMMs were fixed, permeabilized, stained with anti p65 primary antibody, and Alexafluor-488 tagged anti-rabbit secondary antibody, and visualized via fluorescence microscopy (40×). **D.** Image analysis using Metamorph Software was subsequently used to quantify p65 translocation to the nucleus of stimulated 129SvEv and Dectin-1^−/−^ BMMs. **E.** Representative fields of 129SvEv and Dectin-1^−/−^ BMMs 1h after incubation with conidia. Coverslips were incubated with Calcufluor white to detect conidia, and examined by DIC and fluorescence microscopy (conidia are visualized as blue). **F.** Number of conidia per 100 BMM; **G.** Percent BMM with associated conidia. At least 300 cells were examined in each group. Data represent mean +/− SEM of two BMM cultures, and similar results were found in two repeat experiments.

**Figure 6A** shows Syk phosphorylation in 129SvEv BMMs after 15 min incubation, which was sustained for 30 and 60 min. In contrast, p-Syk was not detected in Dectin-1^−/−^ BMMs until 60 min post-exposure. P-Syk was not detected in naïve BMM or after LPS stimulation. Similarly, IκB phosphorylation was elevated in 129SvEv BMMs after 15 min incubation with swollen conidia compared with naïve BMM; however, elevated P-Syk was not detected in Dectin-1^−/−^ BMMs until 60 min incubation (**Figure 6A**). Additionally, *A.fumigatus*-induced CXCL1/KC production by BMMs from 129SvEv mice was significantly higher than BMMs from Dectin-1^−/−^ mice (**Figure 6B**). As shown in **Figure 6C, D**, NF-κB p65 nuclear translocation was apparent in 129SvEv BMM after 30 min and 60 min, but not in Dectin-1^−/−^ BMMs within this time period.

As cell-associated conidia were detected in the p65 translocation experiments, and Dectin-1 – mediated phagocytosis has been shown to inhibit cell signaling [42], we examined the effect of Dectin-1 on cell associated conidia. BMM were incubated with swollen conidia at 100 MOI as described above, then incubated with Calcofluor white to identify conidia [43]. Cell association was examined by DIC and fluorescence microscopy, and quantified by direct counting. As shown in **Figures 6E-G**, Dectin-1^−/−^ BMM had significantly less conidia associated with each macrophage than 129SvEv BMMs (**Figure 6F**). Similarly, the percent of total Dectin-1^−/−^ BMM macrophages with associated conidia was also significantly lower than 129SvEv BMMs (**Figure 6G**).

Together, these data indicate that *A. fumigatus* conidia bind Dectin-1 on resident corneal macrophages, which then stimulates Syk and NFκB–dependent production of CXCL1/KC that is important for neutrophil recruitment to the cornea.

### *A. fumigatus* killing in the cornea is dependent on TLR4, but not TLR2 or MD-2

**Figure 7:**
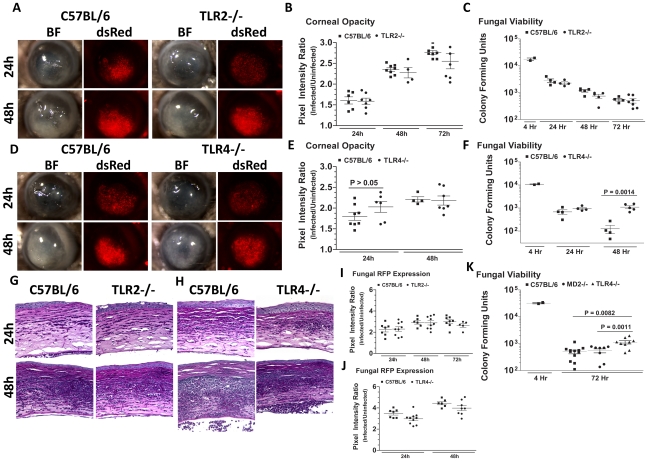
The role of TLR2, TLR4, and MD-2 in *A. fumigatus* keratitis. Corneas of TLR2^−/−^, TLR4^−/−^, MD-2^−/−^, and C57BL/6 mice were infected with *A. fumigatus* conidia as described above. **A.** Brightfield and fluorescence microscopy of corneas from TLR2^−/−^ and C57BL/6 mice infected for 24 and 48h with strain Af293.1RFP. **B.** Corneal opacity quantification and **C.** Fungal viability post-infection in TLR2^−/−^ and C57BL/6 mice. **D.** Brightfield and fluorescence microscopy of corneas from TLR4^−/−^ and C57BL/6 mice infected with Af293.1RFP. **E.** Corneal opacity quantification and **F.** Fungal CFU post-infection of TLR4^−/−^ and C57BL/6 mice. Note increased fungal survival at 48 hr post-infection in TLR4^−/−^ mice. **G.** 5µm PASH stained central cornea sections of infected TLR2^−/−^ and **H.** infected TLR4^−/−^ and C57BL/6 corneas at 24 and 48 h post-infection. **I.** Fungal RFP expression in TLR2^−/−^ and C57BL/6 mice post-infection. **J.** Fungal RFP expression in TLR4^−/−^ and C57BL/6 mice post-infection. **K.** Fungal viability at 4h and 72h post-infection of MD2^−/−^, TLR4^−/−^, and C57BL/6 mice infected with *A. fumigatus* Strain Af-BP. Data are representative of six independent experiments, with five mice per time point.

To determine the role of TLR2 and TLR4 in *Aspergillus* keratitis, C57BL/6, TLR2^−/−^ and TLR4^−/−^ mice were injected intrastromally with Af293.1RFP conidia, and corneal opacification, cellular infiltration, fungal growth and fungal survival were measured as described above. As depicted in **Figure 7A–C**, there were no significant differences between TLR2^−/−^ and C57BL/6 mice in any of these parameters, indicating that TLR2 has no role in corneal infection with these organisms. Similar results were obtained with clinical isolate Af-BP (data not shown).

**Figure 7D, E** illustrate that as with TLR2^−/−^ mice, there were no significant differences in corneal opacification, cellular infiltration or fungal RFP expression between TLR4^−/−^ and C57BL/6 corneas; however, in marked contrast to TLR2^−/−^ mice, significantly more CFU were recovered from TLR4^−/−^ mice after 48h compared with C57BL/6 mice (**Figure 7F**), indicating an impaired ability of TLR4^−/−^ mice to clear the infection, and suggesting a role for TLR4 in fungal killing. Increased CFU was also detected in TLR4^−/−^ corneas infected with the Af-BP clinical isolate at 48h and 72h after infection (**Table 1**), indicating that the role for TLR4 is consistent among Af strains. Despite the difference in CFU, there was no difference in cellular infiltration between TLR4^−/−^ and C57BL/6 and TLR2^−/−^ corneas (**Figure 7G, H**), nor any differences in fungal RFP expression (**Figure 7I, J**).

**Table 1:** C57BL/6 and TLR4^−/−^ mice were infected with 1×10^5^ *A.fumigatus* conidia (Strain Af293.1RFP or Af-BP).

Expt	Fungal Strain	Time	C57BL/6 CFU	SEM	n	TLR4^−/−^ CFU	SEM	n	p value
**1**	Af293.1RFP	48 hr	126	50	4	1113	208	4	0.0014
**2**	Af293.1RFP	72 hr	553	65	4	1144	88	5	0.0178
**3**	Af-BP	72 Hr	495	111	12	1129	278	9	0.0082

At set time-points post-infection, eyes were enucleated and plated on SDA media for CFU analysis. Data from three experiments are shown indicating the time post-infection (Time) in which a difference between C57BL/6 and TLR4^−/−^ mice was observed, colony forming units (CFU), standard error of the mean (SEM), and population size (n) per experiment.

Given the role for TLR4 in fungal viability, we also examined the role of TLR4 co-receptor MD-2, which binds lipid A of Gram negative bacteria. Corneas of C57BL/6, TLR4^−/−^, and MD-2^−/−^corneas were infected with Af293.1RFP, and CFU were quantified after 24, 48, and 72h. As before, CFU recovered from corneas of TLR4^−/−^ mice were significantly higher than C57BL/6 at 72h post-infection **Figure 7K**. However, despite the documented role for MD-2 in responding to LPS, there was no difference in fungal survival in MD-2^−/−^ compared with C57BL/6J mice, indicating that the role of TLR4 fungal killing is MD-2 independent.

### Cellular infiltration and fungal killing is dependent on MyD88, but not TIRAP or TRIF

Given that fungal survival is dependent on TLR4, and TLR4 signaling involves the adaptor molecules MyD88, MAL/TIRAP and TRIF (MyD88: myeloid differentiation primary-response gene 88; TIRAP: toll-interleukin 1 receptor domain containing adaptor protein, which is also called MAL:MyD88-adaptor-like protein; TRIF: TIR-domain-containing adaptor protein inducing IFNβ [44]), we next examined the role of these adaptor molecules in cellular infiltration and fungal killing. C57BL/6, MyD88^−/−^, TIRAP^−/−^ and TRIF^−/−^ mice were injected intrastromally with Af293.1RFP conidia, and markers of infection were examined as before.

**Figure 8:**
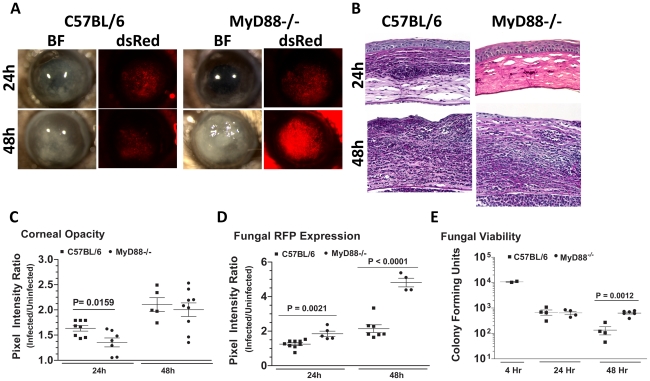
The role of MyD88 in *A. fumigatus* keratitis. C57BL/6 and MyD88^−/−^ mice were infected with Af293.1RFP as before, and examined at 24 and 48h post-infection **A.** Representative images from brightfield and fluorescence microscopy of MyD88^−/−^ and C57BL/6 corneas. **B.** PASH stained 5 µm central corneal sections of infected MyD88^−/−^ and C57BL/6 mice 24h and 48h post-infection. **C.** Corneal opacity quantification, **D.** Fungal RFP expression, and **E.** Fungal viability. Data are representative of two independent experiments, with five mice per time point.

**Figure 8A** shows that MyD88^−/−^ mice had less corneal opacification scores at 24h compared with C57BL/6 mice, but not 48h after infection; conversely, MyD88^−/−^ corneas also had increased fungal RFP (**Figure 8A**). We found impaired cellular infiltration in the corneal stroma of MyD88^−/−^ mice compared with C57BL/6 mice at 24h (**Figure 8B**); however, at 48h, there was an intense cellular infiltration into the MyD88^−/−^ corneas. Image analysis shows significantly lower corneal opacification (**Figure 8C**), but higher fungal RFP expression (**Figure 8D**) in MyD88^−/−^ mice versus C57BL/6 mice. Consistent with the latter observation, **Figure 8F** shows significantly increased fungal CFU at 48h post-infection in MyD88^−/−^mice. These data demonstrate that MyD88 regulates early cellular infiltration and fungal survival in *Aspergillus* keratitis. Further, even though cellular infiltration is detected in MyD88^−/−^mice after 48h, there was no difference in CFU, indicating a role for MyD88 on the ability of neutrophils and infiltrating macrophages to kill *Aspergillus*.

**Figure 9:**
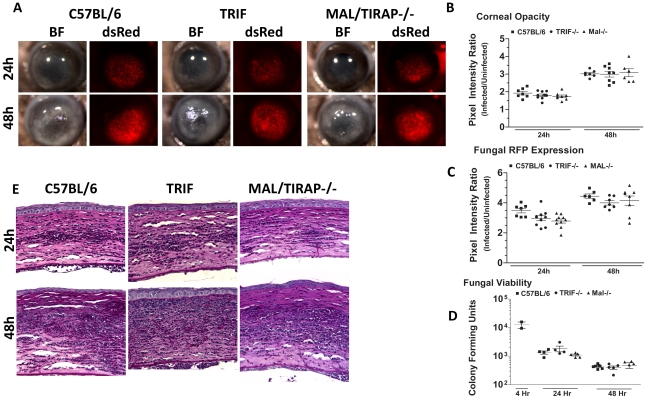
The role of TIRAP and TRIF in *A. fumigatus* keratitis. Corneas of TIRAP^−/−^, TRIF^−/−^, and C57BL/6 mice were abraded and infected with Af293.1RFP. **A.** Representative brightfield and fluorescence microscopy of corneas at 24 and 48h post-infection. **B.** Corneal opacity quantification, **C.** Fungal RFP expression, and **D.** Fungal viability post-infection. **E.** 5µm PASH stained central cornea sections of infected Mal/TIRAP^−/−^,TRIF^−/−^, and C57BL/6 mice post-infection. Data are representative of three independent experiments, with five mice per time point.

To determine if MyD88 is due to TLR4 signaling, we infected mice deficient in TIRAP, which is essential for MyD88 signaling by TLR4. We also infected mice deficient in TRIF, which mediates the TLR4 (and TLR3), MyD88-independent signaling pathway [45]. **Figure 9A** shows that TRIF^−/−^ and TIRAP^−/−^ mice develop corneal opacification that is not different from C57BL/6 mice. Similarly, the presence of dsRed Aspergillus in the corneas of TRIF^−/−^ and TIRAP^−/−^ mice was similar to C57BL/6 mice. Image analysis also showed no difference in opacity or dsRed expression, respectively, between C57BL/6, TRIF^−/−^, and TIRAP^−/−^ mice (**Figure 9B, C**). In addition, there was no difference in fungal CFU among the three strains at either 24h or 48h (**Figure 9D**) or in cellular infiltration among these strains (**Figure 9E**). These findings indicate that although MyD88 has a critical role in *Aspergillus* keratitis, there is no apparent involvement of TIRAP or TRIF.

### IL-1R1 mediates cellular infiltration to the corneal stroma in *Aspergillus* keratitis

**Figure 10:**
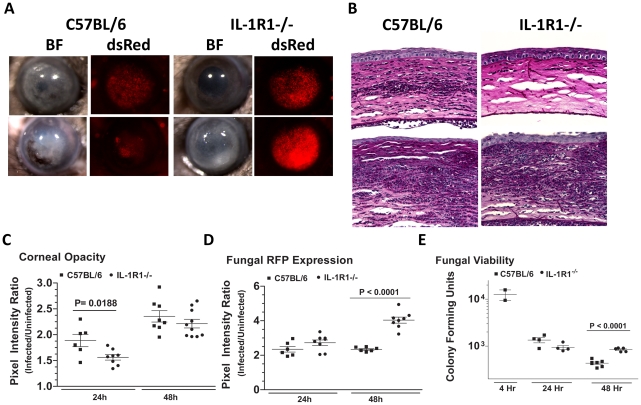
The role of IL-1R1 in *A. fumigatus* keratitis. Corneas of IL-1R1^−/−^ and C57BL/6 mice were infected with Af293.1RFP as before, and examined after 24h and 48h. **A.** Representative brightfield and fluorescence microscopy of corneas from IL-1R1^−/−^ and C57BL/6 mice post-infection. **B.** 5 µm PASH stained central cornea sections of infected IL-1R1^−/−^ and C57BL/6 mice at 24h and 48h post-infection. **C.** quantification of corneal opacity, **D.** Fungal RFP expression, and **E.** Fungal viability. Data are combined from two repeat experiments.

The role of MyD88, but not TIRAP in *Aspergillus* keratitis led us to conjecture that the IL-1 receptor (IL-1R1), which signals through MyD88 independently of TIRAP, could mediate cellular recruitment during *Aspergillus* keratitis. To test this hypothesis, we injected 1×10^5^ *A. fumigatus* conidia into the corneas of C57BL/6 and IL-1R1^−/−^ mice. **Figure 10A** shows that IL-1R1^−/−^ mice had significantly lower corneal opacification and increased fungal RFP expression and survival at 24 and 48h post-infection. Conversely, there was significantly less cellular infiltration into the corneal stroma of IL-1R1^−/−^mice compared with C57BL/6 mice (**Figure 10B**). Image analysis revealed decreased corneal opacity at 24h (**Figure 10C**) and increased fungal dsRed expression at 48h post-infection in IL-1R1^−/−^ mice compared to C57BL/6 mice (**Figure 10D**). Correspondingly, **Figure 10E** shows increased fungal CFU 48h post-infection in IL-1R1^−/−^ mice compared to C57BL/6 mice. Taken together, these observations indicate that IL-1R1 mediates early cellular infiltration and fungal survival in *Aspergillus* keratitis, which is a similar phenotype to MyD88^−/−^ mice.

## Discussion

*Aspergillus* is a major cause of visual impairment and blindness worldwide; however, the nature of the host response to these organisms in the cornea is not well understood. Our findings show that critical components of the innate immune response in the cornea include *c-fms* ^+^ macrophages and dendritic cells in addition to Dectin-1, TLR4, MyD88, and IL-1R1. We also showed that there was no role for MD-2, TLR2, TIRAP or TRIF. Taken together, these observations are consistent with a sequence of events that is initiated by expression of β-glucan on germinating conidia in the corneal stroma, and recognition by Dectin-1 expressed on resident corneal macrophages and dendritic cells. Dectin-1 mediated activation of p-Syk, p-IκB, and translocation of NFκB to the nucleus of these cells results in production of CXCL1/KC and IL-1β within 10h of infection, and recruitment of neutrophils to the corneal stroma. Neutrophils kill *Aspergillus* by attaching to hyphae and releasing cytotoxic proteases, antimicrobial peptides, and reactive oxygen species [46]. Although this inflammatory response causes corneal opacification, the corneas eventually heal, leaving scarified tissue. However, blockade of this response at any of these stages allows the organisms to grow unimpaired, resulting in corneal perforation.

Germinating *Aspergillus* conidia encounter a dense, highly organized matrix in the corneal stroma, with anti-parallel layers of collagen separated by keratan sulfate proteoglycans that are essential for corneal transparency; however, the growing hyphae migrate through the stromal matrix and penetrate the basement (Descemet's) membrane of the corneal endothelium, which forms the barrier to the anterior chamber. Unless killed by neutrophils entering the anterior chamber from iris vessels (seen as a hypopyon in infected individuals), hyphae can penetrate the posterior eye and cause endophthalmitis, at which point enucleation of the infected eye is often indicated.

Although the cornea was long considered to be an immune privileged tissue, it has been well established that macrophages and dendritic cells are resident in the corneal stroma and epithelium [47]–[52]. Recently, a role for macrophages in mediating *Fusarium* and *Candida* keratitis was identified [53]. Similarly, in the present study we identified *c-fms* ^+^ resident macrophages and dendritic cells as essential mediators of cellular recruitment and fungal survival into the cornea during *A.fumigatus* infection, implicating bone marrow derived cells rather than corneal epithelial cells or fibroblasts as the cellular mediators of innate immune recognition [53].

In the current study, we found that β-glucan is not only expressed on *Aspergillus* germinating conidia and hyphae *in vitro* as shown previously [37]–[39], but is also expressed during corneal infection. Furthermore, Syk phosphorylation was detected in infected corneas, and Syk and IκB were phosphorylated in bone marrow macrophages in a Dectin-1 dependent manner. Correspondingly, both NFκB translocation to the nucleus, and CXCL1/KC and IL-1β production were significantly lower in Dectin-1 deficient compared with control macrophages. Similarly, Dectin-1^−/−^ corneas had impaired pro-inflammatory cytokine production and cellular infiltration compared with control corneas. These findings indicate that expression of Dectin-1 on resident corneal macrophages is essential for the initial innate immune recognition of *A.fumigatus*, for the subsequent pro-inflammatory cytokine response, and ultimately for cellular recruitment into the cornea.

We also found that the physical association of swollen conidia with bone marrow macrophages, which includes attachment and phagocytosis, is dependent on Dectin-1 expression. This observation is consistent with reports showing Dectin-1 dependent phagocytosis of β-glucan coated particles [42], [54], and that phagocytosis inhibits Dectin-1 signaling [42], indicating that macrophage interactions with hyphae that are too big to be ingested may have enhanced signaling compared with conidial forms. Our findings also indicate a possible role for Dectin 1 in early killing of swollen conidia and germ tubes rather than hyphae, where we found no differences in β-glucan staining among the *A. fumigatus* strains. Taken together, it is likely that during corneal infection, macrophages and neutrophils utilize Dectin-1 to bind conidia and germ tube developmental stages of *A.fumigatus*, which can be phagocytosed and presumably killed.

Results from the current study are consistent with reports on pulmonary aspergillosis in which germinating *Aspergillus* conidia in the lungs express β-glucan, and Dectin-1 mediates cellular infiltration and fungal killing [38], [39]. Our data are also in agreement with a recent study showing that Dectin-1^−/−^ mice have lower cytokine and chemokine production in the lungs after intratracheal infection, resulting in impaired neutrophil recruitment and increased susceptibility to *Aspergillus* [56]. The role of Dectin-1 in responding to *Aspergillus* therefore appears to be conserved in the cornea and lungs.

Although studies with fungal cell wall components indicate that Dectin-1 and TLR2 collaborate in recognizing β-glucan [57]–[59], we found no role for TLR2 in *Aspergillus* keratitis, indicating that Dectin-1 mediates cellular recruitment independently of TLR2. Additionally, TLR2-independent receptor collaboration occurs in a sequential manner during non-opsonic phagocytosis of *C. albicans* by macrophages, where Dectin-1, CR3, and mannose receptors collaborate in recognition and phagocytosis [60]. In the current study, phagocytosis of swollen *A. fumigatus* conidia was impaired in Dectin-1^−/−^ bone marrow macrophages, raising the possibility that a similar collaboration occurs during macrophage phagocytosis of this pathogen. In support of this notion, the mannose receptor was one of the few genes found to be upregulated in a microarray analysis of *Aspergillus* –infected corneas [61]. Future studies will determine if a similar mechanism occurs for macrophage phagocytosis of *Aspergillus* conidia.

In pulmonary aspergillosis, TLR2 and TLR4 have no role in otherwise immunocompetent animals, although *Aspergillus* CFU were elevated in vinblastin- or cyclophosphamide-treated TLR2^−/−^and TLR4^−/−^ mice [62], [63]. In contrast, we showed increased *Aspergillus* CFU in TLR4^−/−^, but not TLR2^−/−^ mice. Although this is consistent with our earlier observation that TLR4^−/−^ mice have impaired clearance of *Fusarium* in the cornea [64], the mechanism of TLR4 regulation of *Aspergillus* survival has yet to be determined. Given that TLR4^−/−^ mice exhibit defects in fungal killing but not cellular recruitment, we predict that the primary role for TLR4 is on infiltrating neutrophils and macrophages that mediate fungal killing rather than on resident macrophages, which regulate cell recruitment to the cornea. Further, as fungal clearance is unimpaired in MD-2^−/−^ corneas, and MD-2 is the binding site for the lipid A moeity of LPS [65], [66], TLR4 likely recognizes *Aspergillus* hyphae at an MD-2 independent site of the receptor. TLR4 recognizes *C. albicans o*-linked mannosyl residues [59] and *Cryptococcus neoformans* glucuronoxylomannan [67], although the ligand on *Aspergillus* hyphae has yet to be identified. In addition to being the receptor for LPS, MD-2 mediates TLR4 dimerization and cell signaling [44], [68]; therefore, it is possible that TLR4 recognition of the fungal cell wall does not induce signaling. In support of this notion, we found that the absence of TIRAP or TRIF did not affect cellular infiltration or fungal survival. We also noted that although TLR4^−/−^ mice have elevated *Aspergillus* CFU, there was no difference in RFP expression between TLR4^−/−^ and C57BL/6 mice. RFP quantification measures total fungal load, but not viability, and CFU and RFP measurements correlated well in most studies; however, in TLR4^−/−^ mice, more RFP^+^ hyphae appear to be viable, possibly due to production of fungistatic rather than fungicidal mediators in the tissue, including lactoferrin and lipocalin [69].

In the current study, MyD88^−/−^ mice had delayed cellular infiltration and unimpaired fungal growth, which is similar to the role of MyD88 in pulmonary aspergillosis [63], [70], and indicates an essential role for this adaptor molecule in *Aspergillus* keratitis. However, although TLR4 signaling through MyD88 requires the accessory adaptor molecule TIRAP [44], we found no detectable effect on the progression of *Aspergillus* keratitis in the absence of TIRAP. We therefore examined the role of IL-1R1, which signals through MyD88 in the absence of TIRAP [44], and showed that IL-1R1^−/−^ mice have a similar phenotype as MyD88^−/−^ mice. As IL-1β is produced in the cornea early after infection, it seems reasonable to assume that the MyD88 dependence is due to IL-1R1 signaling. In the corneal stroma, IL-1R1 is expressed not only by macrophages and dendritic cells, but also by resident keratocytes in the corneal stroma, which can differentiate into fibroblasts and produce pro-inflammatory and chemotactic cytokines, including CXCL1/KC [71]–[73]. IL-1R1 also mediates the host response in pulmonary candidiasis [63], and we identified a similar role for IL-1R1 and MyD88 in trauma-induced and biofilm-associated *Fusarium* keratitis [64], [74], indicating that the cornea employs similar responses to regulate infection by filamentous fungi.

In conclusion, results of the current studies using a murine model of fungal disease demonstrate essential, though distinct roles for Dectin-1 and TLR4. There also appears to be a role for these receptors in human fungal disease as specific polymorphisms in Dectin-1 and TLR4 genes are associated with susceptibility. A Dectin-1 polymorphism resulting in an early stop codon was associated with reduced β-glucan binding and increased susceptibility to *Candida albicans* infections [75], [76], whereas TLR4 polymorphisms correlate with susceptibility to aspergillosis in recipients of stem cell transplants [77]. Taken together, results from human genetics studies and animal model studies combine to demonstrate that these receptors have an essential role in fungal infection, and are therefore potential targets for immunotherapy that are common to multiple fungal pathogens. Future studies will examine the potential of targeting receptors in prevention and treatment of fungal keratitis.

## Materials and Methods

### Source of mice

All animals were treated in accordance with the guidelines provided in the Association for Research in Vision and Ophthalmology ARVO statement for the Use of Animals in Ophthalmic and Vision Research, and were approved by Case Western Reserve University IACUC. C57BL/6 mice (6–12 wk old), and IL-1R1^−/−^ mice on a C57BL/6 background were purchased from The Jackson Laboratory (Bar Harbor, ME). 129SvEv mice were purchased from Taconic Farms (Hudson, NY). Dectin 1^−/−^ mice on a 129SvEv background were provided by Dr. Gordon Brown, University of Aberdeen, UK, MD-2^−/−^ mice were provided by Dr K. Miyake, University of Tokyo, and TLR2^−/−^, TLR4^−/−^, TRIF^−/−^, and MyD88^−/−^ mice on a C57BL/6 background were provided by Dr. Shizuo Akira, Osaka University, Osaka, Japan, TIRAP^−/−^ mice were provided by Dr Ruslan Medzhitov, Howard Hughes Medical Institute, Yale University, NewHaven, CT. MAcrophage Fas-Induced Apoptosis (Mafia) mice were obtained from Sandra Burnett, Dept. of Microbiology and Molecular Biology, Brigham Young University, Provo, UT [36].

### Fungal strains, media, and growth conditions

*Aspergillus fumigatus* strains used in this study were cultured on Vogel's Minimal Media (VMM) w/wo 4% agar +/− supplementation with 10 mM uracil and 5 mM uridine at 37°C/5% CO_2_ unless otherwise stated. *A. fumigatus* Strain Af-BPis a fungal keratitis clinical isolate from a patient treated at Bascom Palmer Eye Institute (Miami, FL) provided by Dr. Darlene Miller. *A. fumigatus* Strain B-5233 is a clinical strain isolated from the lungs of a patient with severe neutropenia provided by Dr. Kwon-Chung (NIAID) [78]. The uracil auxotroph strain Af293.1 (Δ*pyrG*1) [79] was a gift from Gregory May (M.D. Anderson Health Science Center, Texas). Complementation of *pyrG*1 and constitutive RFP expression by strain Af293.1 was achieved via transformation with the plasmid pRG3AMA1-RFP forming strain Af293.1RFP (Figure S1).

### Construction of the dsRed fluorescent *A. fumigatus* strain Af293.1RFP

All primers used in this study are listed in **Supplementary Figure S1A**. The first step in the construction of Af293.1RFP, entailed PCR amplification of the glyceraldehyde 3 phosphate dehydrogenase promotor (*gpdA*) on the plasmid pDV2 [80] using a forward primer bearing a 5′ Kpn1 overhang (Kgpd-F) and a reverse primer bearing a 5′ complementary DNA sequence to the improved monomeric dsRed fluorescent protein- encoding gene (*rfp*), expressed on the plasmid pMT-RFP (Rgpd-R) [81], [82]. Next, the *rfp* gene on pMT-RFP was PCR amplified using a forward primer bearing a 5′ complementary sequence to the 3′ end of the *gpdA* promotor on pDV2 (Grfp-R) and a reverse primer harboring a 5′ Kpn1 cut site overhang (Krfp-R). Both PCR amplicons (*gpdA* promotor = 1084 Af-BP; *rfp* = 769 Af-BP) underwent fusion PCR, using primers Kgpd-F and Krfp-R to yield a single fusion PCR amplicon *gpdA*:*rfp* (Af-BP). Lastly, Kpn1-digested *gpdA*:*rfp* was ligated to Kpn1-digested pRGAMA-1 yielding an 11.8 kb plasmid (**Figure S1B**). The resulting plasmid, pRG3AMA1-RFP, was transformed into the uracil auxotroph strain, Af293.1Δ*pyr*G according to the method described by May [83]. **Figure S1C** shows that the resulting strain, Af293.1RFP, constitutively expressed dsRED fluorescence (580 nm) at each morphological stage of the organism. Additionally, there was no difference in the growth kinetics of this strain with the parental Af293.1 strain (data not shown).

### Development of a mouse model of *Aspergillus* keratitis

*A. fumigatus* strains were cultured for 2–3 days on VMM without uracil or uridine in 25 cm^2^ tissue culture flasks. Fresh conidia were disrupted with a bacterial L-loop and harvested in 5 ml PBS. Pure conidial suspensions were obtained by passing the culture suspension through sterile PBS-soaked cotton gauze positioned at the tip of a 10 ml syringe. Conidial suspensions were quantified using a haemacytometer, and adjusted to a final concentration of 5×10^4^ conidia/µl in PBS. Mice were anaesthetized by intraperitoneal (IP) injection of 2.25 mg ketamine and 0.45 mg xylazine, and the corneal epithelium was abraded using a 30-gauge needle. Through the abrasion was inserted a 33-gauge Hamilton syringe from which a 2 µl injection containing 1×10^5^ conidia (Optimal inoculum size for induction of keratitis based on preliminary studies; data not shown) was released into the corneal stroma. Mice were examined daily under a stereomicroscope for corneal opacification, ulceration, and perforation. At set time points, animals were euthanized by CO_2_ asphyxiation, and eyes were either placed in 10% formalin and embedded in paraffin and sectioned at 5 µm intervals, or excised and placed in 1 ml of sterile saline and homogenized for quantitative culture. For cyclophosphamide immunosuppression, mice were given 180 mg/kg cyclophosphamide (Sigma-Aldrich) via I.P. injection at days 3 and 1 prior to infection [84]. All animals were bred under specific pathogen-free conditions and maintained according to institutional guidelines.

### Depletion of *c-fms* expressing cells using Mafia mice

MAcrophage Fas-Induced Apoptosis (Mafia) mice are C57BL/6J mice harboring an eGFP and suicide-protein expressing transgene downstream of the macrophage and dendritic cell lineage-specific *c-fms* promoter[36], [85]. As such all MACs/DCs in these mice, constitutively express eGFP and a membrane-bound suicide protein. To deplete *c-fms* expressing cells, Mafia mice received five daily ip injections (10 mg/kg) of the covalently-linked dimer (AP20187; Ariad Pharmaceuticals) followed by two days rest before the experiment. AP20187 has affinity for the FK506 binding protein region of the suicide protein; and homodimerization of the suicide proteins activates the intramolecular cytoplasmic Fas domains, with the subsequent induction of caspase-8 mediated apoptosis in all MACs/DCs. Our recent study showed that this protocol results in loss of viable eGFP+ cells in the cornea [36], [85].

### Imaging corneal opacity and fungal RFP expression

Mice were sacrificed by CO_2_ asphyxiation and positioned in a thee-point stereotactic mouse restrainer. Corneal opacity (Brightfield), fungal proliferation (RFP; 580nm) and cellular infiltration (eGFP; 488nm), were visualized in the intact cornea using a high-resolution stereo fluorescence MZFLIII microscope (Leica Microsystems) and Spot RT Slider KE camera (Diagnostics Instruments). In some experiments, corneas were dissected and examined using an inverted Leica DMI 6000B microscope. All images were captured using SpotCam software (RT Slider KE; Diagnostics Instruments).

### Quantification of corneal opacification, fungal dsRed and *c-fms*+ cell eGFP expression

To quantify corneal opacity objectively, brightfield images of mouse corneas were analyzed using Metamorph software (Molecular Devices). Briefly, a constant circular region encompassing the cornea was defined, and the pixel intensity within this region summed to yield a numerical value, called the pixel intensity corresponding to the total amount of light reflected from the cornea (i.e. opacity). Similarly, fungal dsRed RFP and *c-fms* ^+^ cell eGFP expression were quantified via Metamorph image analysis. All images were obtained with the same Spot RT Slider KE camera using the same Spot Advanced Software under the same magnification, exposure (BF = 0.4s; RFP = 10s; eGFP = 2s), gain (BF = 1; RFP/eGFP = 16), and gamma (BF/RFP/eGFP = 1.85) parameters.

### Quantification of *Aspergillus* colony forming units (CFUs)

For assessment of fungal viability, whole eyes were homogenized under sterile conditions in 1 ml PBS, using the Mixer Mill MM300 (Retsch, Qiagen, Valencia, CA) at 33 Hz for 4 min. Subsequently, serial log dilutions were performed and plated onto bacteriologic-grade Sabouraud dextrose agar plates (Becton Dickenson). Following incubation for 24h at 37°C, the number of CFUs was determined by direct counting.

### Identification of fungi and inflammatory cell recruitment

Eyes were enucleated and fixed in 10% formalin in PBS (Fisher) for 24h. Tissues were then dehydrated in graded ethanol concentrations at room temperature (65% 1×, 80% 2×, 95% 1×, 100% 3×; 1 h for each change of solution), followed by three 1h changes of xylene, and 4 changes of paraffin at 60°C under 15mm Hg vacuum to remove air bubbles. Five µm sections from the center of the cornea (as determined by noncontiguous iris morphology) were cut and stained with Periodic-Acid Schiff (PASH) for identification of fungi and inflammatory cell recruitment.

### Detection of β-glucan expression and neutrophil infiltration during live corneal infection

To detect β-glucan expression during live corneal infection, 5-µm paraffin sections were deparaffinized. Slides were blocked with 1.5% normal rabbit serum in PBS for 1h, then incubated with primary mouse anti-fungal β-glucan IgM (BF-Div; Biothera/Brown Univ.) diluted to 24 µg/ml with 1% BSA (Fisher) for 1h at 37°C. The slides were then washed 3× in PBS plus 0.05% Tween 20 (PBS-T; Sigma) and incubated with alexafluor-488 rabbit-anti-mouse IgM (Invitrogen) diluted to 1µg/ml in PBS for 1h at 37°C. The slides were washed 3× in PBS-T and imaged by fluorescence microscopy (magnification, 400×). A similar process was performed to stain 5µm corneal sections for neutrophils using monoclonal rat-anti-mouse neutrophil IgG (NIMP-R14, AbCam, Cambridge, MA), and alexafluor-488 tagged rabbit-anti-rat IgG (Invitrogen).

### Detection and quantification of β-glucan surface expression by *A. fumigatu*s grown *in vitro*

*A.fumigatus* strains were cultured for 3 days in VMM+4% agar. Pure conidial suspensions were prepared from the 3-day culture, and 5 million conidia were added to 50 ml SDA broth in vented-cap 250 ml tissue culture flasks. The conidia were grown at 37°C/5% CO_2_ for 0, 6, and 10h. At indicated timepoints, *A.fumigatus* was fixed with 4% paraformaldehyde for 30 min, washed 3× with PBS and spun onto Superfrost microscope slides (Fisher) using a Cytospin centrifuge. Subsequently, slides were blocked with 1.5% normal rabbit serum in PBS for 1h, then incubated with primary mouse anti-fungal β-glucan IgM (BF-Div; Biothera/Brown Univ.), diluted to 24 µg/ml with 1% BSA (Fisher) for 1 h at 37°C. The slides were then washed 3× in PBS-T and incubated with alexafluor-488 rabbit-anti-mouse IgM (Invitrogen) diluted to 1µg/ml in PBS for 1h at 37°C. The slides were washed again 3× in PBS-T and imaged by fluorescence microscopy (magnification, 400×). Subsequently, images were analyzed using Metamorph software and the numerical output of average 488nm pixel intensity/area of fungal cells was used to quantify fungal β-glucan surface expression. To minimize the confounding variable of enhanced fluorescence due to cell clumping, only 488nm fluorescence emanating from isolated fungal cells was used for quantitation.

### Detection of pSyk, IκB, and Dectin-1 via Western blot analysis

Cornea protein extracts were prepared by homogenization in cell lysis buffer (Cell Signaling) plus 1mM phenylmethylsulphonyl-fluoride (PMSF) using a Tissue-Lyser (described above). Similarly, cell culture protein extracts were prepared via lysis in cell-lysis buffer+PMSF. Total protein was quantified via Bicinchoninic Acid Assay (Pierce), denatured with 2× Laemmli buffer (Sigma) and heated to 95°C for 5 min. 20 µg total protein was loaded onto each well of a 10% polyacrylamide gel (BioRad), separated via electrophoresis, and transferred to nitrocellulose. Blots were stained accordingly with anti-Dectin-1 (RandD; MAB1756), anti-phosphoSyk (Cell Signaling; 2710), anti-pIκB (Cell Signaling; 2859), and anti-β-actin (Cell Signaling; 4967). HP-tagged secondary antibodies were purchased from Santa Cruz Biotechnologies. All blots were developed with GE Healthcare ECL Western Blotting Detection Reagent (Amersham) or Supersignal West Femto Maximum Sensitivity Substrate (Pierce).

### Intracellular NF-κB localization and cell-associated conidia in bone marrow derived macrophages (BMM)

Mice underwent euthanasia by CO_2_ asphyxiation, and femurs and tibias from 129SvEv and Dectin-1^−/−^ mice were removed, cleaned, and centrifuged at 5000×g for 45 s at 4°C. Any contaminating red blood cells were lysed in 5 ml RBC Lysis Buffer (eBioscience), and remaining bone marrow cells were cultured in bacteriologic grade petri dishes in 6 ml Macrophage Growth Medium (MGM:DMEM w/L- Glutamine, Na-Pyruvate, HEPES,10% FBS, P/S, 30% L929 cell conditioned medium). On day 5, and every 2 days thereafter, the cell supernatant was aspirated, and fresh MGM media was added. Adherent cells were harvested between 7–14 days of culture, and counted. 2×10^4^ cells were cultured onto sterile 18mm^2^ coverslips (Corning) in a 6 well-plate, and treated with LPS (100 ng/ml; positive control), or 6h *A.fumigatus* strain Af-BP swollen conidia (MOI = 100) for 15, 30 and 60 min. Following activation, BMM were fixed with 4% paraformaldehyde for 15 min at room temperature, permeabilized using 0.1% Triton X-100 in PBS for 1 min at RT, and incubated with rabbit anti-mouse p65 (1∶100; eBioscience Ltd) in PBS containing 10% goat serum for 1h at RT. Coverslips were washed 2× with PBS and cells were incubated with Alexa Fluor 488-labeled goat anti-rabbit IgG antibody (Molecular Probes Inc.) in PBS at RT for 1h and washed 2× with PBS. The cells were mounted on glass slides using Vectashield mounting medium with DAPI (Vector Laboratories, UK), and examined by fluorescence microscopy, and average pixel intensity (green fluorescence) per nucleus (DAPI) was calculated using Metamorph.

For cell association studies, bone marrow macrophages incubated with *A.fumigatus* strain Af-BP swollen conidia were washed and fixed in 4% paraformaldehyde as described above, then incubated 5 min with Calcufluor white (Sigma) at a 1∶1 ratio with 10% KOH. After washing 2× with PBS, cells were examined by DIC and fluorescence microscopy, and associated conidia per 100 cells, and the percent cells with associated conidia was determined after direct examination of at least 300 cells per coverslip. Two coverslips were examined, and the mean and SD were calculated.

### Cytokine assays

Corneas were homogenized in 150 µl Reagent Diluent (R & D Systems, Minneapolis, MN) using the Retsch MM 300 ball miller at 33 Hz for 4 min (Qiagen). For analysis of bone marrow macrophage cytokine production, cell culture supernatants were obtained and assayed directly. Half-well cytokine assays were performed using Duoset ELISA kits (R & D Systems) according to the manufacturer's directions.

### Statistical analysis

Statistical analysis was performed for each experiment using an unpaired t test (Prism, GraphPad Software). A p value<0.05 was considered significant.

## Supporting Information

Figure S1 An improved monomeric dsRED RFP expressing *A. fumigatus* strain was developed to visualize *A.fumigatus* during live tissue infection.
**A.** Primers used in construction of pRG3AMA1-RFP are listed. GTAC = Kpn1 recognition cut site; *Italicized* = DNA complementary to *rfp* on RgpdR and DNA complementary to *gpdA* on GrfpR **B.** The plasmid pRG3AMA1-RFP harbors *rfp* downstream of the constitutive Glyceraldehyde 3 phosphate dehydrogenase promotor (*gpdA*), allowing constant visualization of *A.fumigatus* under fluorescence microscopy **C.** Af293.1RFP shows similar morphological developmental progression from conidia→swollen conidia→germ tube→hyphae→mycelial mass as the parental strain Af293.1 Insets shows micrographs of the individual morphological growth stages of *Aspergillus fumigatus*.
(2.51 MB TIF)

Figure S2 **A.** Normal mouse cornea showing epithelium (**epi**), corneal stroma, corneal endothelium (**endo**) and anterior chamber. **B.** 580 nm fluorescence image of naïve mouse cornea showing no background emission in the RFP spectrum.
(3.77 MB TIF)
