# Supplementary figures and images for: Distinct Roles for Dectin-1 and TLR4 in the Pathogenesis of Aspergillus fumigatus Keratitis
Source: PLoS Pathog. 2010 Jul 1;6(7):e1000976. doi: 10.1371/journal.ppat.1000976 (PMC2895653; doi:10.1371/journal.ppat.1000976)

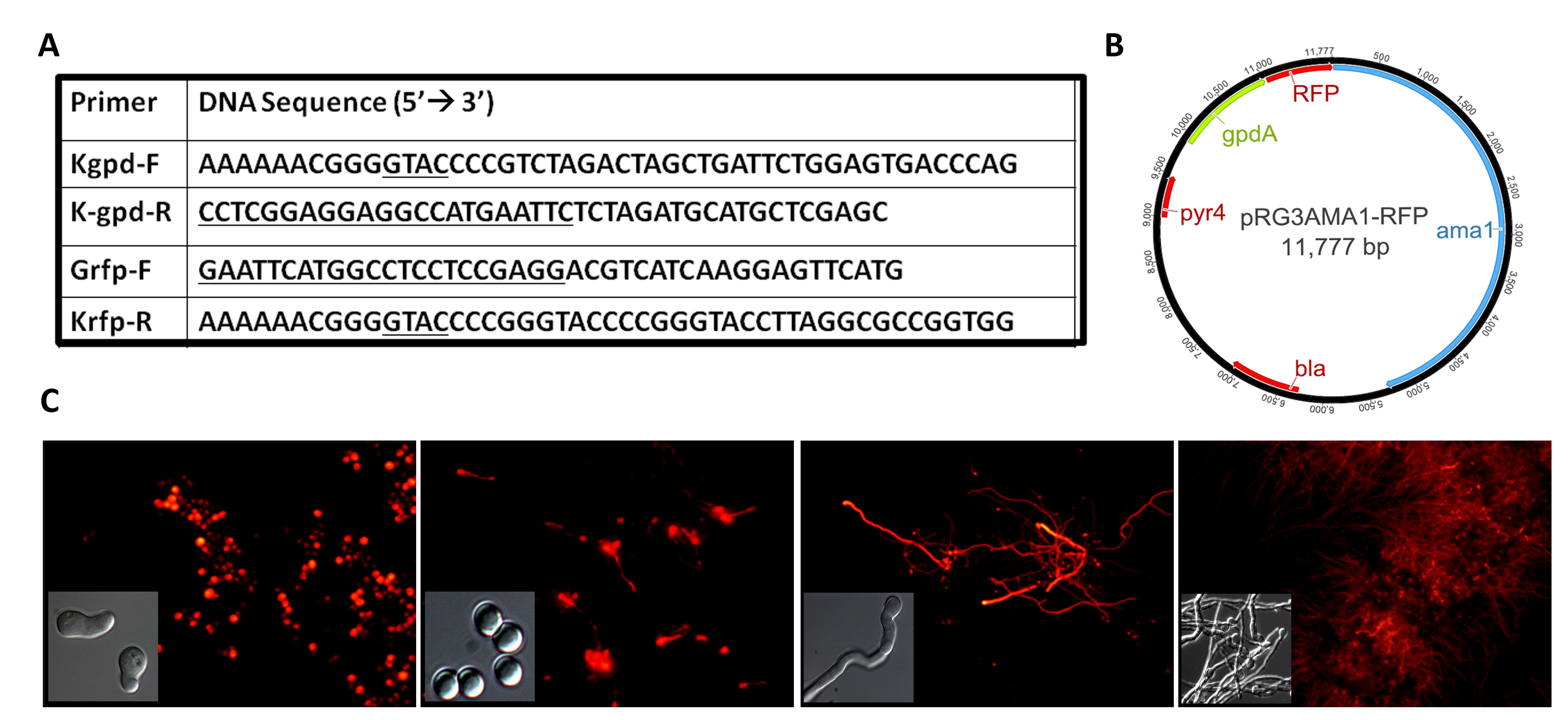

Supplement: Figure S1 — An improved monomeric dsRED RFP expressing A. fumigatus strain was developed to visualize A.fumigatus during live tissue infection. A. Primers used in construction of pRG3AMA1-RFP are listed. GTAC = Kpn1 recognition cut site; Italicized = DNA complementary to rfp on RgpdR and DNA complementary to gpdA on GrfpR B. The plasmid pRG3AMA1-RFP harbors rfp downstream of the constitutive Glyceraldehyde 3 phosphate dehydrogenase promotor (gpdA), allowing constant visualization of A.fumigatus under fluorescence microscopy C. Af293.1RFP shows similar morphological developmental progression from conidia→swollen conidia→germ tube→hyphae→mycelial mass as the parental strain Af293.1 Insets shows micrographs of the individual morphological growth stages of Aspergillus fumigatus. (2.51 MB TIF) [file ppat.1000976.s001.tif]

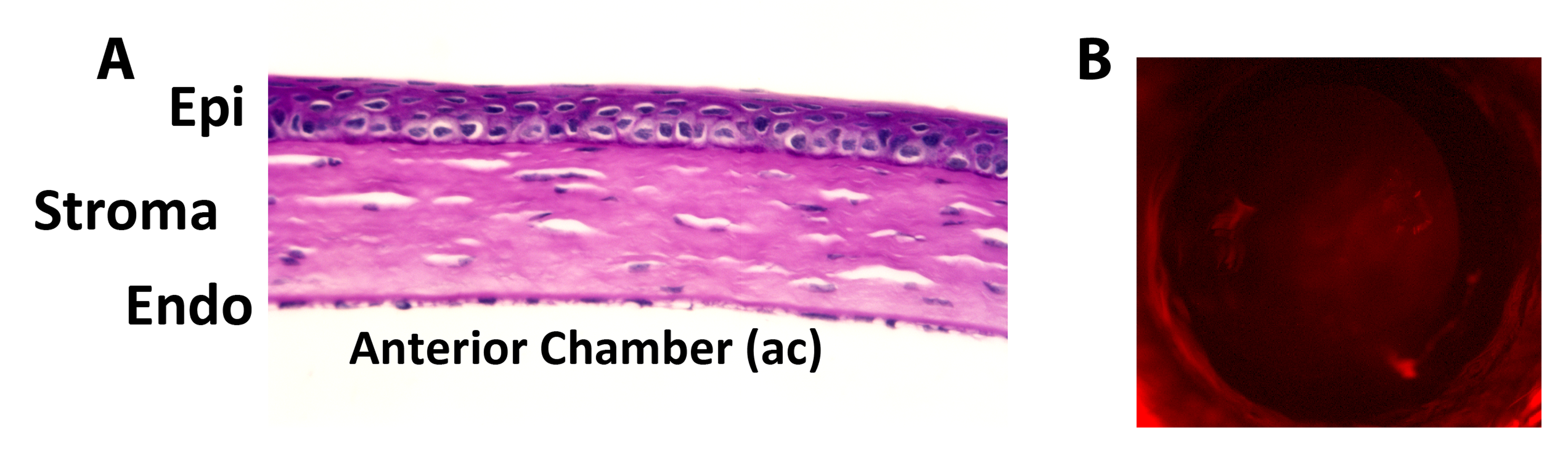

Supplement: Figure S2 — A. Normal mouse cornea showing epithelium (epi), corneal stroma, corneal endothelium (endo) and anterior chamber. B. 580 nm fluorescence image of naïve mouse cornea showing no background emission in the RFP spectrum. (3.77 MB TIF) [file ppat.1000976.s002.tif]
